# Resveratrol Upregulates Cardiac SDF-1 in Mice with Acute Myocardial Infarction through the Deacetylation of Cardiac p53

**DOI:** 10.1371/journal.pone.0128978

**Published:** 2015-06-08

**Authors:** Wang Hong, Shimosawa Tatsuo, Wang Shou-Dong, Zhang Qian, Hou Jian-Feng, Wang Jue, Jin Chen, Qian Hai-Yan, Yang Yue-Jin

**Affiliations:** 1 Center for Cardiac Intensive Care, Beijing Anzhen Hospital, Capital Medical University, Beijing, China; 2 Department of Clinical Laboratory, Faculty of Medicine, The University of Tokyo, Tokyo, Japan; 3 Institute of Chinese Materia Medica, China Academy of Chinese Medical Sciences, Beijing, China; 4 State Key Laboratory of Cardiovascular Disease, Fuwai Hospital and Cardiovascular Institute, Chinese Academy of Medical Sciences and Peking Union Medical College, Beijing, China; Indiana University School of Medicine, UNITED STATES

## Abstract

**Aims:**

We previously demonstrated that resveratrol (RSV) administration causes cardiac stromal cell-derived factor (SDF)-1 upregulation and can enhance the mobilization of stem cells in mice with acute myocardial infarction (AMI). However, the upstream signal transduction involved in SDF-1 regulation in the setting of AMI and RSV administration remains unclear. Because RSV is a sirtuin 1 (SIRT1) activator and SIRT proteins act as deacetylases, we investigated the role of SIRT1 in SDF-1 upregulation and its subsequent effects.

**Methods and Results:**

*In vitro* experiments with H9C2 cardiomyocytes under hypoxia and serum-deprivation conditions showed that p53 acted upstream of SDF-1. RSV could not regulate SDF-1 effectively after SIRT1 silencing, indicating that it is dependent on SIRT1. Subsequently, male C57BL/6 mice were divided into four groups: 1) sham, 2) MI, 3) MI+RSV, and 4) MI+RSV plus nicotinamide, an inhibitor of the deacetylase activity of SIRT (MI+RSV+NAM). Compared with the sham mice, AMI caused a slight increase in the cardiac p53 level and resulted in significant SIRT1 downregulation and p53 acetylation or activation. Compared with the MI mice, MI+RSV administration improved the cardiac SDF-1 level and reversed the reduction of SIRT1 and the activation of p53. Furthermore, we observed less cardiac dysfunction in MI+RSV mice and determined that NAM abolished the effects of RSV.

**Conclusions:**

RSV enhances cardiac SDF-1 excretion after AMI partially through a SIRT1 normalization/p53 inactivation pathway.

## Introduction

Heart failure develops in a substantial number of patients with acute myocardial infarction (AMI), despite early reperfusion therapy. Stem cell-based therapies and chemokine- or cytokine-based therapies are promising procedures for myocardium regeneration and the attenuation of ventricular remodeling, fibrosis or dysfunction [1, 2]. The stromal cell-derived factor (SDF)-1/CXCR4 (the receptor of SDF-1) axis has been demonstrated to be critical for the recruitment of stem cells to the ischemic area. A higher level of SDF-1 in the injured myocardium improves cardiac repair [2–4].

Many studies have shown that SDF-1 is upregulated or released spontaneously in the infarct and peri-infarct regions in AMI. The autologous SDF-1 level peaks 24–72 h after AMI and becomes normalized seven days after AMI [5, 6]. However, this natural modulation is not sufficient for the induction of functional recovery in many AMI models or patients [5]. SDF-1 delivery by a virus vector, plasmid or direct injection into the injured tissue early after AMI has been proven to be more effective [5, 7, 8], but these methods are not convenient or feasible in clinical therapy. The development of an oral medicine that could be an alternative to these methods may allow a markedly easier and safer application.

Resveratrol (RSV) is a component of the polyphenol extract of grape skin/seed, red wine and the root of *Polygonum cuspidatum* [9]. There are seven members in the mammalian sirtuin (SIRT) protein family, the many beneficial effects of RSV are mediated by SIRT proteins. It should be said that most researches are focus on SIRT1, which is an essential deacetylase and can regulate the acetylation of various proteins, such as peroxisome proliferator-activated receptor gamma co-activator-1 and p53 [9–11], a transcriptional activator of apoptosis. The inhibition of p53 may increase the concentration of local vascular endothelial growth factor [12]. In oncology research, p53 deletion causes the upregulation of SDF-1 and accelerates the migration of CXCR4^+^ tumor stem cells or tumor development [13, 14].

We attempted to administer atorvastatin and RSV to a murine model of AMI and found that short-term RSV administration can increase cardiac SDF-1 expression and improve the mobilization of very small embryonic-like stem cells (over 80% of which are CXCR4^+^) to the injured heart [15]. However, the role of SIRT1 in RSV induced SDF-1 regulation has not been fully elucidated. In the present study, we aimed to investigate how RSV increases cardiac SDF-1 in AMI and determined that p53 inactivation through its deacetylation by RSV mediates SDF-1 regulation.

## Methods and Materials

### Animals and materials

The study was approved by the Institutional Animal Care and Use Committee of Fuwai Hospital and Cardiovascular Institute. All of the research protocols conformed to the guiding principles for animal experimentation articulated by the Ethics Committee of the Chinese Academy of Medical Sciences and Peking Union Medical College. The authors of this manuscript have certified that they comply with the ARRIVE guidelines, and all efforts were made to minimize suffering [16]. Male C57BL/6 mice, 8–10 weeks of age, were housed in an accredited institute facility in accordance with the institutional animal care policies. For the *in vivo* experiments used to evaluate the effect of SIRT, the mice were randomly divided into four groups: 1) a sham operation group, 2) a myocardial infarction (MI) group, 3) an MI+RSV (25 mg/kg/day in drinking water, from five days before AMI to two days after AMI as described previously [15]) group, and 4) an MI+RSV+nicotinamide (NAM, a SIRT inhibitor, 75 mg/kg/day i.p. by osmotic pump, from five days before AMI to two days after AMI) group. The dose of NAM administered was selected according to a previous report [17, 18]. The mice were sacrificed two days after AMI or four weeks after AMI. The osmotic pump used was purchased from Durect Co. (Alzet 1007D, Cupertino, CA, USA). RSV and NAM were purchased from Sigma (St. Louis, MO, USA).

### AMI model

The mice were anesthetized with an i.p. of 2% chloral hydrate (2 ml/100 g), endotracheally intubated by tracheotomy, and mechanically ventilated using the Inspira Advanced Safety Ventilator (ASV, NP 55–7059, Harvard Corp., Evansville, WI, USA), which supplied 0.75 ml of room air/oxygen 110 times per minute. The heart of each mouse was exposed via a left thoracotomy. After removing the pericardium, the left anterior descending coronary artery (2 mm below the tip of the left auricle) was occluded with an 8.0 Prolene suture (ETHICON, Inc., Somerville, NJ, USA). Occlusion was confirmed by observing the LV pallor immediately.

### *In vitro* experiments

H9C2 cells were purchased from ATCC (Manassas, VA, USA) and incubated in DMEM (Hyclone Laboratories, Logan, UT, USA) with 10% vol/vol FBS and 1% vol/vol antibiotics at 37°C in a humidified atmosphere of 5% CO_2_. To determine whether p53 activity affects the cardiac SDF-1 level and investigate the specific effect of SIRT1, p53 and SIRT1 were silenced. p53 and SIRT1 siRNAs were obtained from Santa Cruz Biotechnology (Santa Cruz, CA, USA) and transfected into the cells using the corresponding agents (Santa Cruz Biotechnology) according to the manufacturer’s protocol. The control cells (treated with the transfection reagents only) and the cells transfected with siRNA were treated in serum-free medium and then incubated in a sealed hypoxic GENbox jar fitted with a catalyst (Bio-Me´rieux, Marcy l'Etoile, France) to scavenge free oxygen for 24 h. The oxygen tension in the medium was measured using an air indicator (Bio-Me´rieux). After hypoxia, the cells were collected and subjected to western blotting.

### Western blotting

After the mice were sacrificed two days after AMI via cervical dislocation, the cells from the bone marrow (BM, at least three samples in each group) and the LVs (n = 5–7 in each group) were isolated after drawing blood. The protein in the total cell lysates, including H9C2 cardiomyocytes, was extracted by pipetting the cells or homogenizing the tissues in RIPA buffer with a proteinase inhibitor cocktail (Roche Diagnostics, Basel, Switzerland). Thirty to forty micrograms of protein were separated on SDS-polyacrylamide gels, transferred to PVDF membranes, and incubated with primary antibodies overnight at 4°C. Anti-SDF-1 (Santa Cruz Biotechnology) antibody was used for evaluating the SDF-1 level. The nuclear protein fractions were isolated using commercially available kits (BioVision, Mountain View, CA, USA) and immunoblotted with anti-p53 (Cell Signaling Technology), anti-acetyl-p53 K379 (K382 human protein, Cell Signaling Technology) (tissue samples), anti-acetyl-p53 K370 (K373 human protein, Millipore, Billerica, MA, USA) (tissue samples), and anti-SIRT1 (Cell Signaling Technology, Inc., Beverly, MA, USA for tissue samples, Abcam Inc., Cambridge, MA, USA for cell samples) antibodies. After washing and incubation with secondary antibodies, the signals were visualized with the ECL substrate (Pierce Biotechnology), quantified with the Image Scan software (Scion Image; Scion Co.), and standardized to the expression of β-actin (Sigma) and nucleophosmin (NPM, Sigma, for nuclear proteins).

### ELISA

SDF-1α is an important isoform of SDF-1. To inspect the changes in SDF-1α, the LVs were isolated and maintained at -80°C until use. The tissue SDF-1α was measured with a mouse ELISA kit (RayBiotech Inc., Norcross, GA, USA) according to the manufacturer’s instructions as described previously [15].

### Echocardiographic and pathological evaluations

1. **Echocardiographic assessment.** Transthoracic echocardiography was performed (n = 4–9 in each group) at the end of the fourth week. The mice were anesthetized with an i.p. of 2% chloral hydrate (2 ml/100 g), maintained in the decubitus position and allowed to breath spontaneously during the procedure. Transthoracic echocardiography was performed with a 35-MHz phased-array ultrasound system (VisualSonics Inc., Toronto, Canada). M-mode tracings of the LVs were recorded at the papillary muscle level to measure the interventricular septal dimension (IVS), LV end-diastolic dimension (LVEDD) and LV end-systolic dimension (LVESD). The ejection fraction (EF) was calculated automatically.
2. **Pathological studies at the end of the fourth week.** After the mice were sacrificed via cervical dislocation after echocardiography, the LVs were obtained. At least three LVs per group were fixed with 4% paraformaldehyde, embedded in paraffin and cut into 3-μm-thick sections (three sections of each LV at the papillary muscle level). Masson-trichrome (MT) staining was performed to quantify the extent of fibrosis in the LVs. The fibrotic area and total area of the LV on each image were measured using the Image-Pro-Plus software (Media Cybernetics), and the fibrotic area was calculated as a percentage to the total LV area.

### Statistical analysis

All of the values are expressed as the means ± SEMs. Unpaired Student's *t* test was used for comparisons between two groups. For multiple comparisons, ANOVA followed by Scheffé’s method was used. A value of *p* < 0.05 was considered significant.

## Results

### Effect of p53 and SIRT1 silencing/RSV on SDF-1 regulation in cardiomyocytes under hypoxic conditions

**Fig 1:**
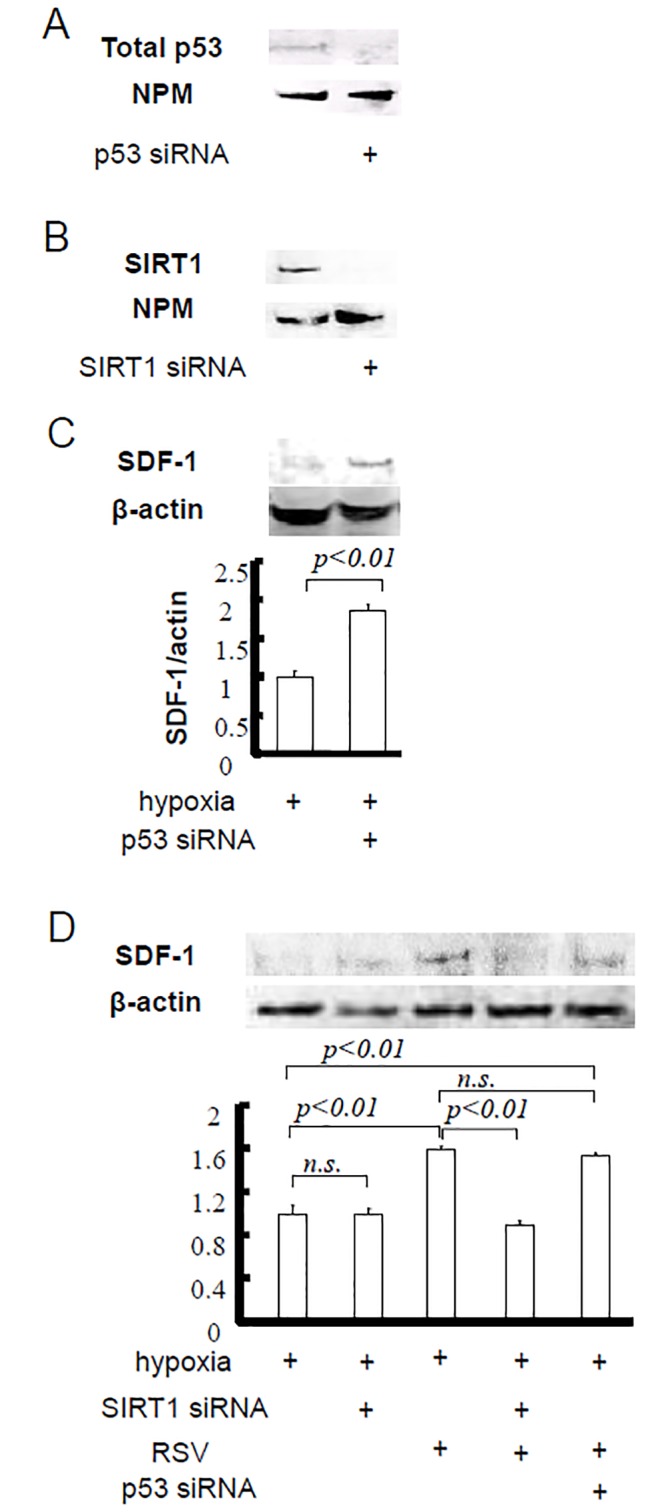
The role of p53 on cardiac SDF-1 and the specific effect of SIRT1 under hypoxia. H9C2 cells were utilized in this experiment. A. p53 siRNA transfection caused p53 silencing. B. SIRT1 siRNA transfection caused SIRT1 silencing. C. p53 silencing increased the SDF-1 level under hypoxia. D. siRNA transfection on the SDF-1 expression or the effect of RSV.

H9C2 cardiomyocytes were transfected with p53 or SIRT1 siRNA, and successful transfection caused a significant downregulation of p53 or SIRT1 (Fig 1A and 1B). The cells were then treated with hypoxia and serum deprivation. The SDF-1 level after hypoxia in the cells transfected with p53 siRNA (1.87±0.07) was higher than that observed in the cells without siRNA transfection (1.00±0.09, Fig 1C). The SDF-1 levels in the cells administered RSV (15 μM) and the cells transfected with p53 siRNA were highest among all of the cells after hypoxia (1.59±0.03 and 1.52±0.03 respectively). SIRT1 siRNA transfection did not inhibit SDF-1 expression when compared with control (hypoxia only), but inhibited the effect of RSV. RSV did not further enhance the SDF-1 expression after p53 silencing (Fig 1D).

### Drug loading affected SIRT1 expression in AMI

**Fig 2:**
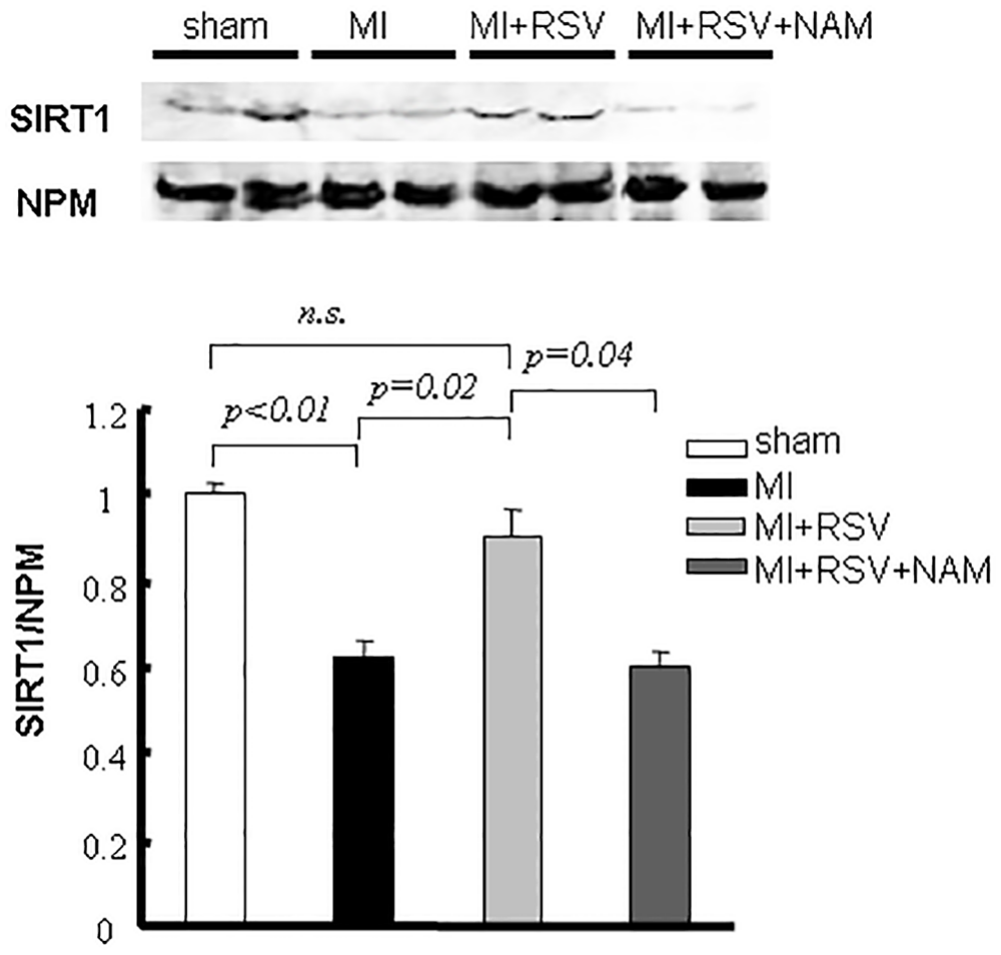
Changes of SIRT1 by MI, RSV and SIRT inhibition. The SIRT1 level in the nuclear extracts (LV) decreased during acute injury. RSV or NAM loading modulated SIRT1 expression in AMI.

The amount of nuclear SIRT1 was investigated by western blotting. The levels of nuclear SIRT1 decreased during acute injury (MI: 0.62±0.04; sham: 1.00±0.03). RSV loading induced a recovery in the nuclear SIRT1 level compared with MI (0.90±0.06), whereas NAM inhibited the effect of RSV (0.60±0.03, Fig 2).

### SIRT status mediated the modulation of p53

**Fig 3:**
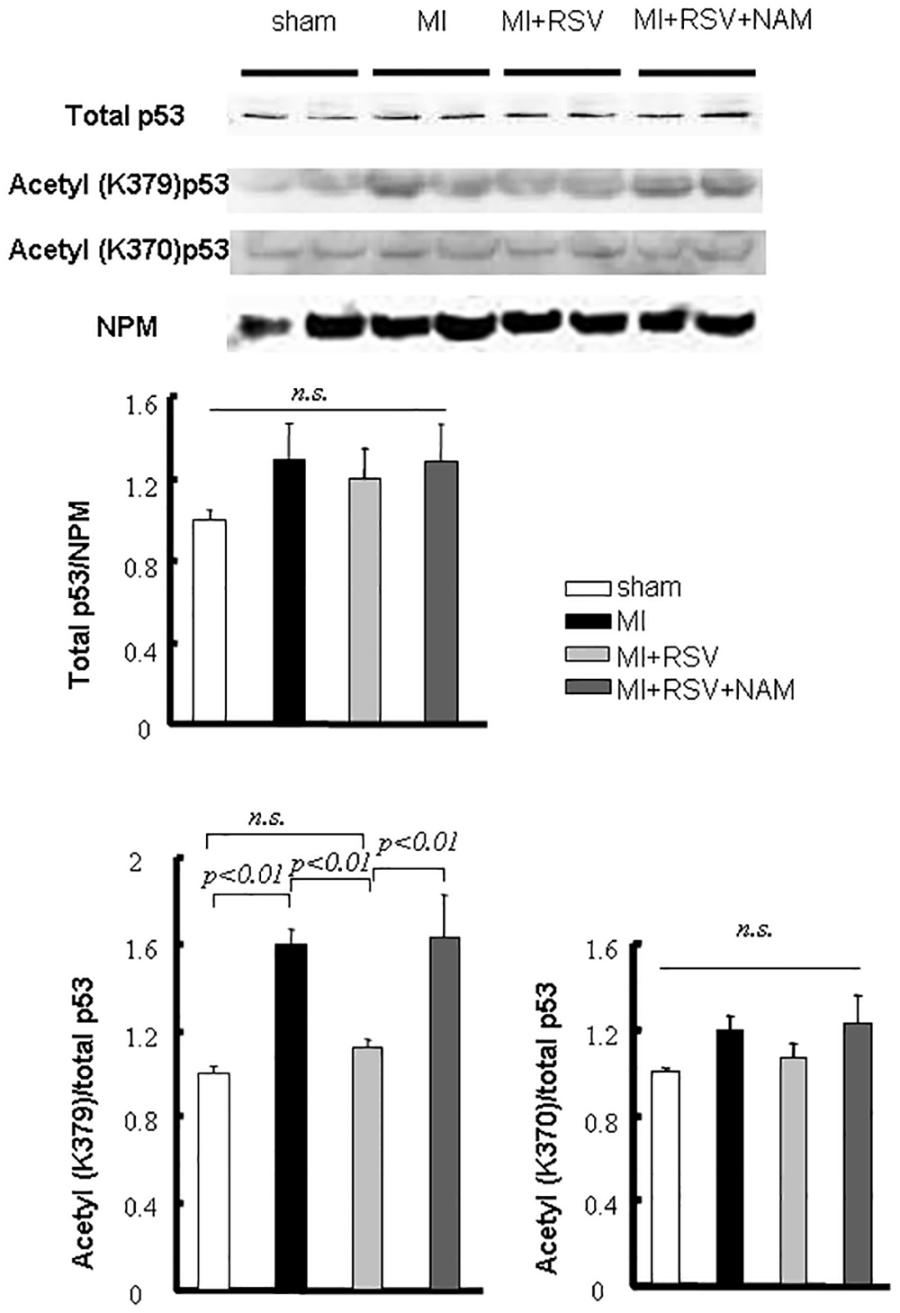
Modulation of p53 caused by RSV. Representative immunoblots and bar graphs of the total p53 and acetyl-p53 (K379 and K370) levels in nuclear extracts (LV). AMI activated p53, and RSV reversed this effect through p53 deacetylation.

The amount of total p53 increased after AMI, but a significant difference was not found among the groups. Mouse p53 acetylation was evaluated at K379 and K370. p53 was acetylated at K379 early after AMI compared with the sham group. The acetylation in the MI+RSV mice (1.12±0.03) was less than that in the MI mice (1.59±0.07), and the effect of RSV could be eliminated by NAM administration (K379 acetylation in MI+RSV+NAM mice: 1.61±0.20). We did not find significant acetylation at K370 (Fig 3).

### The increase in cardiac SDF-1 was dependent on SIRT

**Fig 4:**
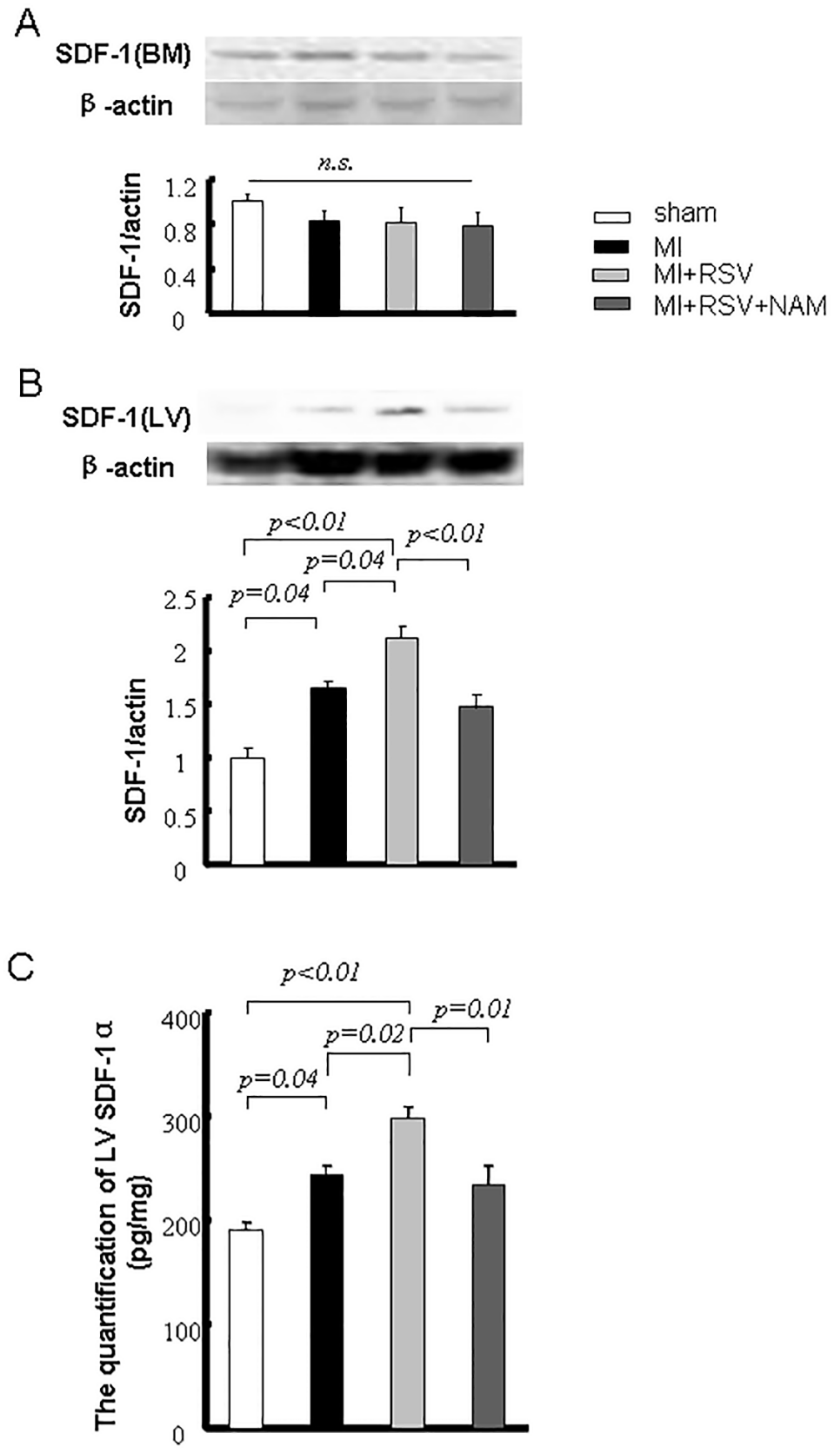
SDF-1 evaluation in the BM and LV. Immunoblots of SDF-1 in BM. B. Immunoblots of SDF-1 in LV. The bar graph shows the quantification of the bands. C. Quantification of LV (infarct and peri-infarct areas) SDF-1α by ELISA.

We did not detect an obvious difference in BM SDF-1 among the groups (Fig 4A). In contrast, compared with the sham mice, the cardiac SDF-1 expression level was increased in the MI mice. The MI+RSV group exhibited the highest cardiac SDF-1 expression level, and this level in the MI+RSV+NAM group was reduced to the level observed in the MI group (Fig 4B). To confirm these results, we evaluated the level of SDF-1α in each group by ELISA. The ELISA results (sham: 191.67±6.26 pg SDF-1α/mg cardiac tissue; MI: 243.07±9.15 pg/mg; MI+RSV: 297.12±13.11 pg/mg; MI+RSV+NAM: 233.64±18.53 pg/mg) were consistent with the western blotting results (Fig 4C).

### SIRT status was parallel to the cardiac function

**Fig 5:**
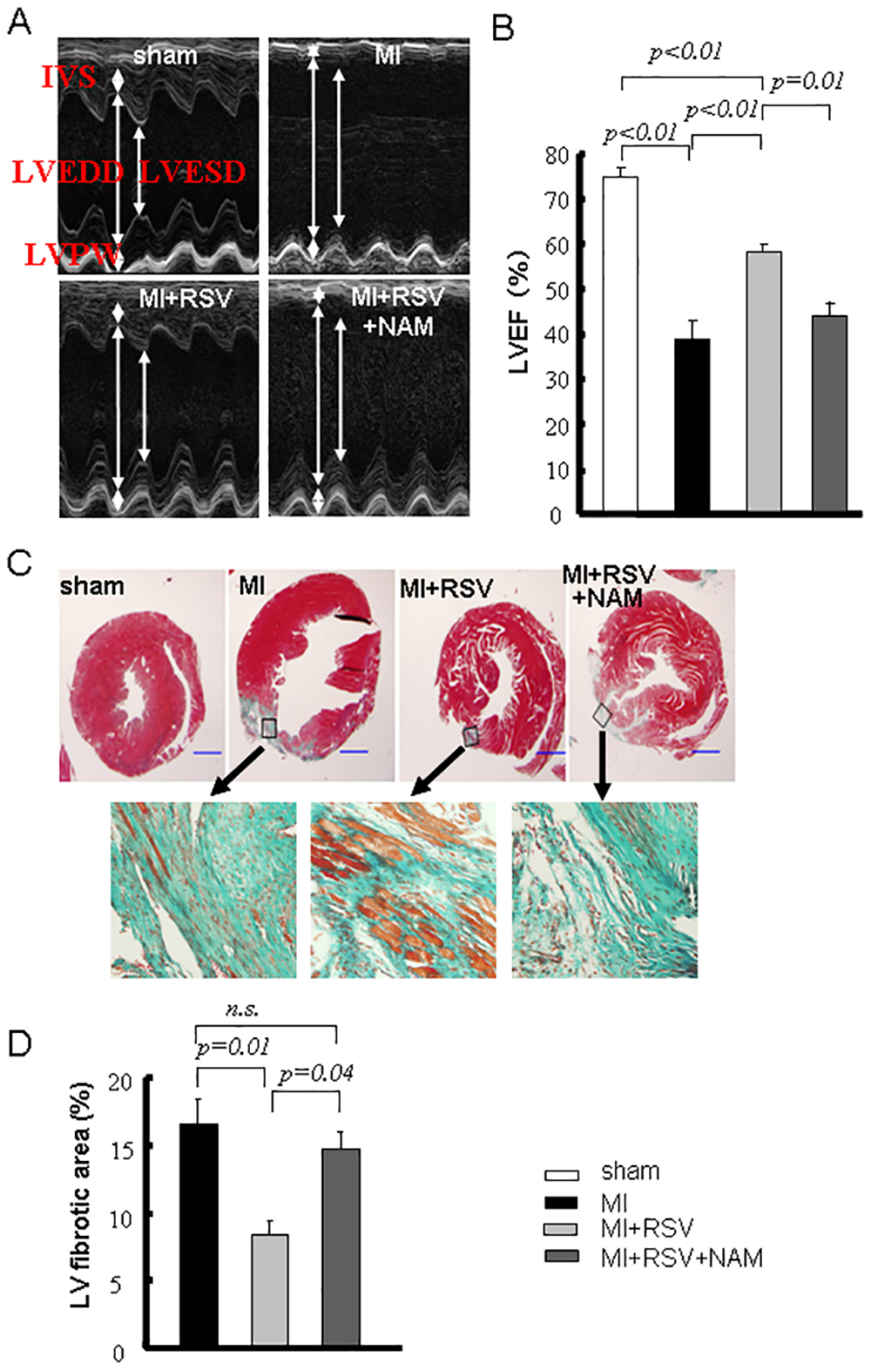
Evaluations of cardiac function and fibrosis. A. Representative photos of M-mode echocardiography at the end of the fourth week. B. Bar graphs for LVEF at the end of the fourth week. C. Representative photos of MT staining at the end of the fourth week. The fibrotic tissue was stained blue. Higher magnifications of the boxed areas illustrate that greater amounts of the underlying myocardium are present within scar tissue in the RSV group. D. Quantification of the fibrotic area. The magnification is ×40 (scale bar = 1 mm).

At the end of the fourth week, the IVS, LVEDD and LVESD in the MI group were significantly different from the corresponding values in the sham group (*p*<0.01 for each). RSV improved LVESD (*p*<0.01 vs. MI), but the improvements in IVS and LVEDD (*p*>0.05 vs. MI) at the end of the fourth week were not significant (Fig 5A, Table 1). The sham mice had an average LVEF of 75.10%. The MI mice had an average LVEF of 39.30%, and this level was elevated to 57.91% by RSV. The LVEF in the MI+RSV+NAM mice (43.98%) was similar to that of the MI mice (*p*>0.05 vs. MI and *p*<0.05 vs. MI+RSV, Fig 5B). The heart rate during the echocardiographic examination showed no difference among the groups (Table 1). Furthermore, the MI mice presented a larger infarct size or severe fibrosis (fibrotic area/the total LV area×100% = 16.74±1.78%). RSV attenuated this effect to some extent (8.39±1.10%), and NAM abolished the effects of RSV (14.77±1.20%, Fig 5C and 5D).

**Table 1:** Parameters used for echocardiographic assessment.

	sham	MI	MI+RSV	MI+RSV+NAM
IVS (mm)	0.46±0.04	0.29±0.02*	0.34±0.02**	0.27±0.01*
LVEDD (mm)	4.76±0.11	5.45±0.09*	5.08±0.07**	5.35±0.08*
LVESD (mm)	2.98±0.09	4.60±0.21*	3.80±0.11*, #	4.41±0.14*, §
LVPW (mm)	0.67±0.06	0.51±0.03	0.61±0.05	0.52±0.03
Heart Rate (bpm)	387.50±14.70	388.11±15.95	381.67±12.93	409.00±17.40

The values are expressed as the means ± SEMs.

LVPW: left ventricular posterior wall; bpm: beats per minute.

* p<0.01 vs. the sham group;

** p<0.05 vs. the sham group;

^#^ p<0.01 vs. the MI group;

^§^ p<0.05 vs. the MI+RSV group.

## Discussion

In the present study, we showed that RSV can promote an increase in the SDF-1 level in infarcted LV early after AMI through SIRT1 upregulation/p53 inactivation. To the best of our knowledge, this study provides the first demonstration of the interaction of RSV/p53 with SDF-1 modulation in AMI.

First, we silenced p53 in cardiomyocytes and found that p53 is upstream of SDF-1 under hypoxia/serum deprivation, which is similar to the results obtained in tumor cells [13, 14]. Second, we found that cardiac SIRT1 was reduced early after AMI and that SIRT1 activation by RSV enhanced the expression of SIRT1, similarly to the results of previous studies [19–21]. Third, p53 activity is regulated at both the transcriptional and post-translational levels, e.g., by acetylation [21–25]. Acetylation enhances p53 binding to target genes and causes transcription of the target [19, 26]. As a mark of p53 activation, the K379 and K370 acetylation of p53 have been well investigated in various cells or models [19, 21–26]. Although we used the nuclear protein fractions of the whole LVs for the evaluation of p53, which may result in similar cardiac p53 levels in sham and MI mice, we clearly showed that p53 is highly acetylated (K379) early after AMI, and this hyperacetylation was reversed by RSV. Furthermore, NAM attenuated the effect of RSV on the post-translational modulation of p53 in the MI model. These results indicate that SIRT1 is upstream of p53 and that the signal transduction affected the SDF-1 level in the injured myocardium.

The activation of p53 contributes to apoptosis, and p21 (CIP1/WAF1) is one of the targets of p53. However, previous reports have shown that p53 hyperacetylation is not accompanied by increased p21 under ionizing radiation and that p21 inhibits the integration of STAT with the STAT-binding site within the SDF-1 promoter and directly obstructs the expression of SDF-1 during arterial wound repair [27, 28]. Therefore, p21 appeared to not be involved in signal transduction. The mechanism through which p53 inactivation causes SDF-1 translation or expression in AMI requires further research. In a cutaneous wound model, p53 silencing enhances the secretion of chemokines, including SDF-1, and improves wound healing [12]. The results support the data obtained in the current study, suggesting that the activation of p53 limits the increase in SDF-1 during injury and that targeting p53 is not the only way to inhibit apoptosis but also may cause regeneration. In parallel, we found that greater levels of the myocardium were present in scar tissue four weeks after AMI in the RSV group.

In the current study, ischemia was the primary reason responsible for the increase in the expression of SDF-1. We surmise that hypoxia-induced factor 1 (HIF-1) induced by MI may be one of the factors involved in the regulation of SDF-1. However, HIF-1 increases p53 expression, and p53 downregulates SDF-1 transcription [12, 14]. Further investigations are required to elucidate the mechanism.

One of the limitations of the current study was that we did not obtain data to support or exclude the possibility whether other SIRT families are involved or not in the pathway. We also did not evaluate the SIRT1 activity. Moreover, RSV has multiple protective effects related to cardiovascular diseases [9]. SDF-1 also contributes to cardiac repair by augmenting other cytokines, chemokines or hormones [4, 29, 30]. Therefore, we cannot conclude that the function improvement observed in the RSV-loaded mice was completely due to SDF-1 upregulation or SDF-1/CXCR4 interaction. We should use both SDF-1 blockade and SDF-1/CXCR4 interferent or label the stem cells to evaluate the role of stem cell recruitment in future research.

In conclusion, RSV activates SIRT1, decreases the activity of p53 via deacetylation, and increases the SDF-1 gradient at the site of cardiac injury. These observations may have clinical importance because they imply another beneficial biological effect of RSV for repair of the infarcted myocardium.
